# Systemic multiomics evaluation of the therapeutic effect of *Bacteroides* species on liver cirrhosis in male mice

**DOI:** 10.1128/spectrum.05349-22

**Published:** 2023-10-11

**Authors:** Ye Rin Park, Hae Lee Lee, Ji Ye Hyun, Jieun Choi, Ji Hyun Moon, Byung Yong Kim, Seung-Jo Yang, Je Hee Lee, Byoung Kook Kim, Tae-Sik Park, Ki Tae Suk, Do Yup Lee

**Affiliations:** 1 Department of Agricultural Biotechnology, Seoul National University, Seoul, South Korea; 2 Institute for Liver and Digestive Diseases, Hallym University, Chuncheon, South Korea; 3 R&D Center, Chong Kun Dang Healthcare, Seoul, South Korea; 4 R&D Discovery Center, CJ Bioscience, Inc, Seoul, South Korea; 5 Chong Kun Dang Bio Research Institute, Gyeonggi-do, South Korea; 6 Department of Life Science, Gachon University, Sungnam, South Korea; 7 Center for Food and Bioconvergence, Research Institute for Agricultural and Life Sciences, Interdisciplinary Programs in Agricultural Genomics, Seoul National University, Seoul, South Korea; 8 Department of Food and Animal Biotechnology, Seoul National University, Seoul, South Korea; 9 Green Bio Science & Technology, Bio-Food Industrialization, Seoul National University, Gangwon-do, South Korea; 10 Kimchi Functionality Research Group, World Institute of Kimchi, Gwangju, South Korea; Korea University, Seoul, South Korea

**Keywords:** liver cirrhosis, *Bacteroides*, multiomics, gut-liver axis, metabolomics, microbiome

## Abstract

The human gut microbiome is engaged in biological homeostasis in the gut-liver axis and across multi-organs. The aim of this study is to investigate the therapeutic effects of human gut-derived microbes, *Bacteroides* species on liver cirrhosis in a mouse model. The experiment was performed on male mice, which were divided into five groups: normal control (NC), disease control, *Bacteroides dorei-*, *Bacteroides cellulosilyticus-*, and ursodeoxycholic acid-supplemented groups after 3,5-diethoxycarbonyl-1,4-dihydrocollidine treatment. The therapeutic effect was evaluated based on liver physiology and the expression level of hepatic fibrosis. Untargeted and targeted metabolic profiling was conducted on cecal, fecal, liver, and serum samples using ultra-performance liquid-chromatography coupled with high-resolution mass-spectrometry. The gut microbial taxonomic composition was analyzed by 16S rRNA gene amplicon sequencing from the stool of each mice group. The *Bacteroides* treatment improved the liver/body weight ratio and normalized hepatic fibrosis biomarkers, including COL1A1. The fecal metabolome showed the most distinctive and characteristic profiles according to different treatments, compared to other sample matrices (cecum, liver, and blood). Key metabolites were identified, which indicated the potential therapeutic effect of the *B. dorei* treatment. Among them, a short-chain fatty acid, propionic acid, showed consistent upregulation in the cecum and liver after the *B. dorei* treatment. Microbiome analysis showed that *Akkermansia muciniphila* was retained in the group treated with *B. dorei* at a similar level as in the NC group. Our current multiomics study of systemic dynamics demonstrated that *Bacteroides* species, particularly *B. dorei*, ameliorated liver cirrhosis by modulating the metabolic and microbial environment to the normal state within the gut-liver axis.

**IMPORTANCE:**

The human gut microbiome mediates bidirectional interaction within the gut-liver axis, while liver diseases, including liver cirrhosis, are very closely related to the state of the gut environment. Thus, improving the health of the gut-liver axis by targeting the intestinal microbiota is a potential therapeutic approach in hepatic diseases. This study examines changes in metabolomics and microbiome composition by treating bacteria derived from the human gut in mice with liver cirrhosis. Interorgan-based multiomics profiling coupled with functional examination demonstrated that the treatment of *Bacteroides dorei* pertained to protective effects on liver cirrhosis by normalizing the functional, metabolic, and metagenomic environment through the gut-liver axis. The study provides the potential value of a multiomics-based and interorgan-targeted evaluation platform for the comprehensive examination and mechanistic understanding of a wide range of biologics, including gut microbes. Furthermore, the current finding also suggests in-depth future research focusing on the discovery and validation of next-generation probiotics and products (postbiotics).

## INTRODUCTION

Chronic liver disease and cirrhosis are diseases that cause mortality rates of approximately 2 million people worldwide each year (1). Common causes are HBV (chronic hepatitis B virus) and HCV (hepatitis C virus) infection, ALD (alcoholic liver disease), NAFLD (nonalcoholic fatty liver disease), and others. Although HBV vaccination and viral hepatitis treatment programs have reduced the incidence of cirrhosis due to viral infection in many countries, liver diseases due to environmental factors, genetic factors, and immune imbalances such as unbalanced diet and excessive alcohol consumption are still a serious problem.

Liver diseases, including liver cirrhosis, are very closely related to the state of the gut environment, and studies on the gut-liver axis support this. The gut and liver communicate through the systemic circulation through the biliary tract and portal vein (2). The liver has an effect on the gut by releasing various bioactive mediators, such as bile acid, into systemic circulation through the bile duct. In the gut, the host and microorganism metabolize endogenic substrates (e.g., bile acid, amino acid) and exogenous substrates (e.g., diet, environmental exposure), and the products move to the liver through the portal vein and affect the liver function.

In liver cirrhosis, it is possible to observe bacterial overgrowth and alteration of the gut microbiota composition (3). This is the result of change in the bile flow and composition, and intestinal immunity imbalance. The alteration of gut microbiota composition causes subsequent epithelial barrier damage (e.g., “leaky gut”), causing bacterial translocation and the systemic circulation of pathogen-associated molecular patterns such as lipopolysaccharide (4). In comparison, numerous studies have shown that microbiota homeostasis relies on metabolic products (e.g., postbiotics). In general, postbiotic components are known to play an implicit and protective role in the intestinal barrier function (5). Postbiotics protect gut tissues by acting on immune cells and increasing the release of anti-inflammatory cytokines (e.g., IL-10). In addition, the expression of tight junction proteins such as zonula occludens is increased, and the function of the epithelial tight junction structure is improved, thus restoring the gut barrier function.

Among the methods presented above, probiotics administration is one of the most useful tools to recover from gut dysbiosis. While early probiotics’ studies focused on nutritional functions such as food or diet supplements, recent probiotics studies focused on therapeutic functions. Despite some advantages (e.g., safety), inconsistent effects or even harmful influences of probiotics have been reported in some cases (6). For example, lactic acid bacteria such as *Bifidobacterium, Lactobacillus, Leuconostoc,* and *Weissella* are known probiotics and can be targets of potential pharmabiotics. However, no significant reduction of these lactic acid bacteria is observed in the gut microbiome of patients with alcohol cirrhosis (Fig. S1B). In this regard, recent efforts focus on the exploration of the next-generation probiotics (NGPs), which are mostly derived from human commensals, and the comprehensive elucidation of the mode of action. *Bacteroides* species are commensal bacteria, occupying a large portion of the mammalian gastrointestinal microbiota (7). It is also notable that *Bacteroides* genus was reduced significantly in the gut microbiome of patients with alcohol cirrhosis (Fig. S1C). In particular, the *Bacteroides dorei* strain is categorized as an NGP, which are gut microbes that exhibit a positive association with health but requires a further in-depth evaluation of beneficial effects and safety before human applications (8).

Our current study evaluated the potential therapeutic effect of two *Bacteroides* species, *Bacteroides dorei* and *Bacteroides cellulosilyticus,* on liver cirrhosis based on the 3,5-diethoxycarbonyl-1,4-dihydrocollidine (DDC) mouse model. The DDC mouse model was used to induce chronic cholestatic liver fibrosis in mice. The DDC model recapitulates the disruption of pathways known to be affected in liver fibrosis (9) (e.g., inhibition of liver-specific transcription factors such as HNF4A, HNF1A, and RXRA). Ursodeoxycholic acid (UDCA)-treated group was used as the positive control regarding the treatment effect in our study. Ursodeoxycholic acid is known to have a positive effect on hepatic steatosis and fibrosis by improving liver enzymes and lipid profiles (10). UDCA is currently the first-line therapy for primary biliary cirrhosis. However, between 30% and 40% of patients do not respond to this therapy, so research for a therapeutic replacement of this drug is needed (11).

The purpose of this study is to examine the effect in terms of changes in metabolomics and microbiome composition by treating bacteria derived from the human gut in a mouse derived from liver cirrhosis in a DDC model. Key metabolites and microbiota distributed in stool, liver, cecum, and serum samples were investigated to find the factors suggesting the improvement of liver health, and ultimately to confirm the preventive effect of human gut microbiota-derived *Bacteroides dorei* and *Bacteroides cellulosilyticus* on liver cirrhosis.

## RESULTS

### Protective effects of *Bacteroides* (*B. dorei, B. cellulosilyticus*) in DDC diet-induced liver fibrosis

**Fig 1:**
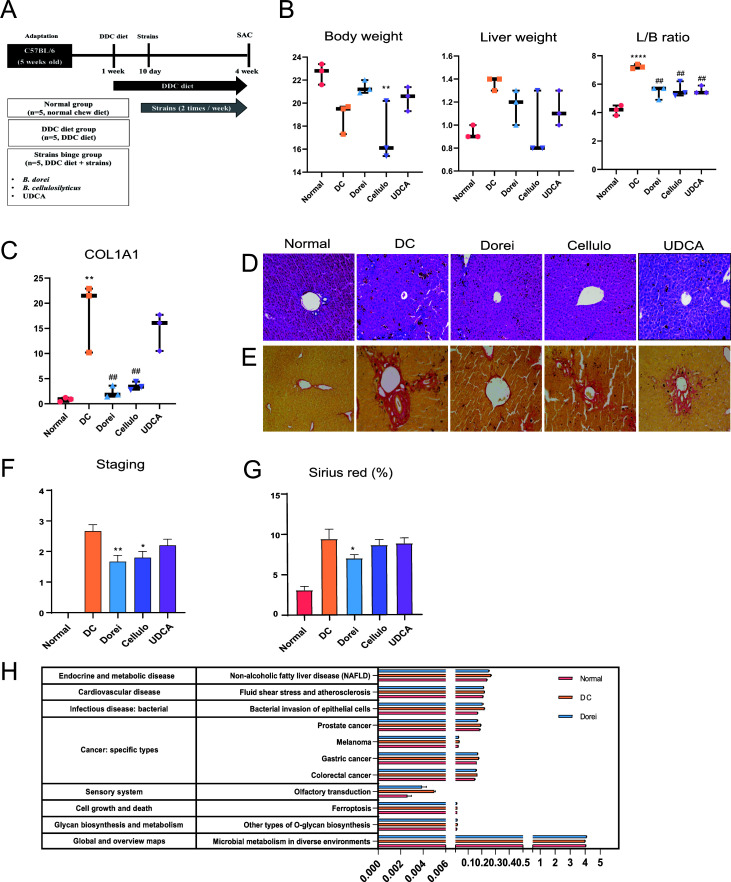
Protective effect of the strain-supplementation and UDCA on DDC diet-induced liver fibrosis. (**A**) Experimental scheme. (**B**) Analysis of body weight, liver weight, and L/B ratio. (**C**) mRNA expression levels of hepatic genes associated with inflammation (one-way ANOVA, followed by Tukey’s *post hoc* test between normal and other groups; **P < 0.01, ****P < 0.0001, one-way ANOVA, followed by Tukey’s *post hoc* test between DC group and other groups ##*P* < 0.01). (**D**) Hematoxylin and eosin staining, and (**E**) sirius red staining of liver sections. (**F**) Pathological effects of strains on the liver based on the clinical research network scoring system (0: no fibrosis, 1: portal fibrosis, 2: periportal fibrosis, 3: septal fibrosis, and 4: cirrhosis). (**G**) Measurement area of sirius red staining. The statistical power is estimated based on one-way ANOVA, followed by Tukey’s *post hoc* test between the DC group and treatment groups using GraphPad Prism 9 (*P < 0.05 and **P < 0.01). (**H**) Functional biomarker analysis based on linear discriminant analysis effect size analysis. The first and second columns indicate the KEGG pathway level 2 and level 3, respectively. *X*-axis indicates the LDA score (log 10). Statistic power is estimated by Kruskal–Wallis test implemented in EzBioCloud server (https://www.ezbiocloud.net).

During the 7-day acclimatization period, C57BL/6J male mice had free access to a normal diet and water. Thereafter, the mice were provided with a DDC diet for 3 days as the acclimatization period. From day 10, 10^9^ CFU of bacterial strains (*B. dorei* and *B. cellulosilyticus*) were orally administered twice a week for 3 weeks (Fig. 1A). L/B ratio (%) increased with a DDC diet (7.2 ± 0.1) (*P* < 0.0001) but decreased after intake of *B. dorei* (5.7 ± 0.3), *B. cellulosilyticus* (5.4 ± 0.3), and UDCA (5.4 ± 0.2) (*P* < 0.01) (Fig. 1B). Expression of hepatic fibrosis markers was confirmed in the liver. In particular, COL1A1 expression level increased significantly when fed with a DDC diet (21.5 ± 4.0), but Dorei (1.8 ± 0.7) and Cellulo (3.4 ± 0.5) decreased to Normal levels (*P* < 0.01) (Fig. 1C). The median level of total bilirubin in the serum increased in the DC group compared to the NC group and recovered moderately in the treatment groups (Fig. S3A). There was no significant difference in TGF-β, an HSC activation marker, and IL-10, an anti-inflammation marker (Fig. S3B). The histopathology of mouse liver shows that DDC-induced liver fibrosis was reduced in Dorei, Cellulo, and UDCA groups (Fig. 1D, E, and G). In accordance with the clinical research network scoring system (12), the staging level of liver fibrosis decreased significantly in Dorei (*P* < 0.01) and Cellulo (*P* < 0.05) groups (Fig. 1F).

Analysis of Kyoto Encyclopedia of Genes and Genomes (KEGG) pathway of functional biomarkers in mouse microbiome (Kruskal–Wallis *H* test) showed that the pathways related to specific types of cancer and sensory system were increased in the DDC diet group compared to normal diet group and decreased in Dorei group (*P* < 0.05) (Fig. 1H).

### Abundance of SCFAs is affected by DDC diet-induced liver cirrhosis and supplement treatment

Short-chain fatty acids (SCFAs) can either be excreted in the feces or taken up by the gut epithelium, entering the portal circulation where they can be metabolized by the liver. Although many *in vitro* experiments demonstrated the beneficial effects of SCFAs (e.g., butyrate for the maintenance of gut-barrier function), some human studies reported the association of fecal SCFA concentrations with excess adiposity (13). In this regard, a more comprehensive profiling of SCFAs is important for the better understanding of the intricate interplay between the gut microbiome and host metabolic homeostasis in healthy or unhealthy environments.

**Fig 2:**
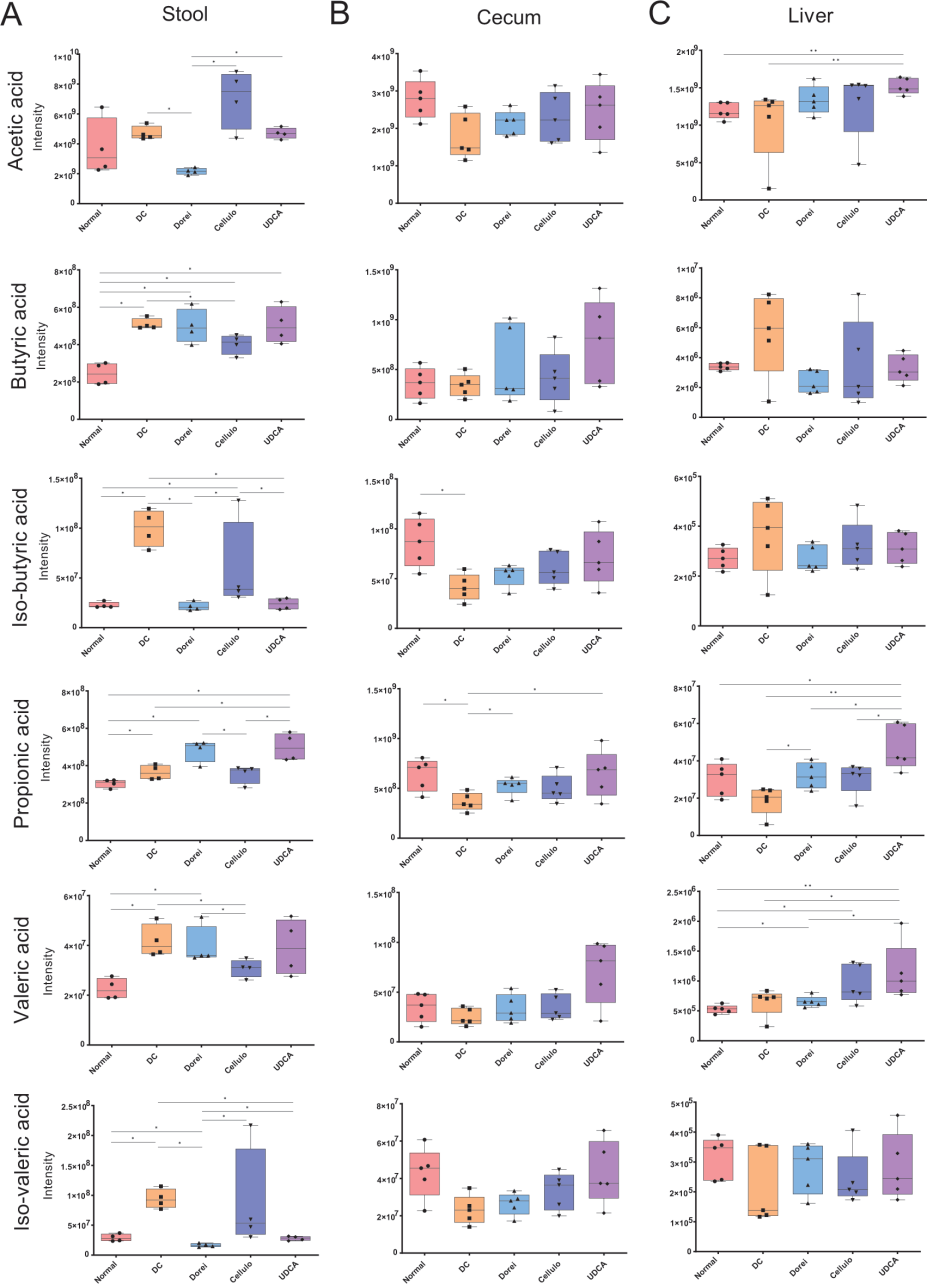
Relative abundances of short-chain fatty acids (acetic acid, butyric acid, iso-butyric acid, propionic acid, valeric acid, and iso-valeric acid) in (**A**) stool, (**B**) cecum, and (**C**) liver. Statistical comparison is performed based on Kruskal–Wallis test using ggpubr function in ggplot2 package (version 0.4.0). Box-and-whisker plot is visualized using GraphPad Prism 9 (*P < 0.05 and **P < 0.01).

Accordingly, the targeted analysis of SCFAs was performed on stool, cecum, and liver samples. In stool samples, all SCFAs except acetic acid were significantly upregulated in the DC group compared to the NC group (Fig. 2A). Relative to the DC group, the *B. dorei*-treated group retained the lower levels in acetic acid, iso-butyric acid, and iso-valeric acid. *B. cellulosilyticus*-treated group presented a significant reduction in butyric acid and valeric acid compared to the DC group, while UDCA-treated group featured significantly lower levels of iso-butyric acid and iso-valeric acid.

In contrast to stool samples, the cecal SCFAs were present in lower levels in the DC group compared to the NC group (Fig. 2B). Iso-butyric acid and propionic acid were substantially downregulated in the DC group. In the *B. dorei*- and UDCA-treated groups, propionic acid was sustained to the NC levels. The hepatic SCFAs showed more heterogenous patterns (Fig. 2C). The NC group did not show significant differences, compared to the DC group. Propionic acid was significantly upregulated in the Dorei group compared to the DC group. Acetic acid, propionic acid, and valeric acid were at significantly higher levels in the UDCA group compared to the NC and DC groups, respectively.

Given our current experimental setting, *B. dorei* treatment significantly increased propionic acid in cecum and liver samples. Similarly, cecal and hepatic propionic acids were at significantly higher levels in the UDCA-treated group. The alteration would be associated with the specific effect rather than the normalized effect since the levels in the liver were significantly higher in the UDCA group, respectively, compared to both DC (*P* < 0.01) and NC (*P* < 0.05) groups. We observed an opposite pattern of SCFAs between stool and cecum samples in both NC and DC groups. In cecum samples, the decreased SCFAs in the DC group compared to the NC group recapitulated that SCFAs are commonly abundant in a healthy gut environment. On the contrary, the increased levels of fecal SCFAs in DC groups implied that SCFAs are more actively excreted in an unhealthy gut environment.

### Global metabolomic profiles of stool and liver demonstrate the most distinct discrimination among NC, treatment groups, and DC

**Fig 3:**
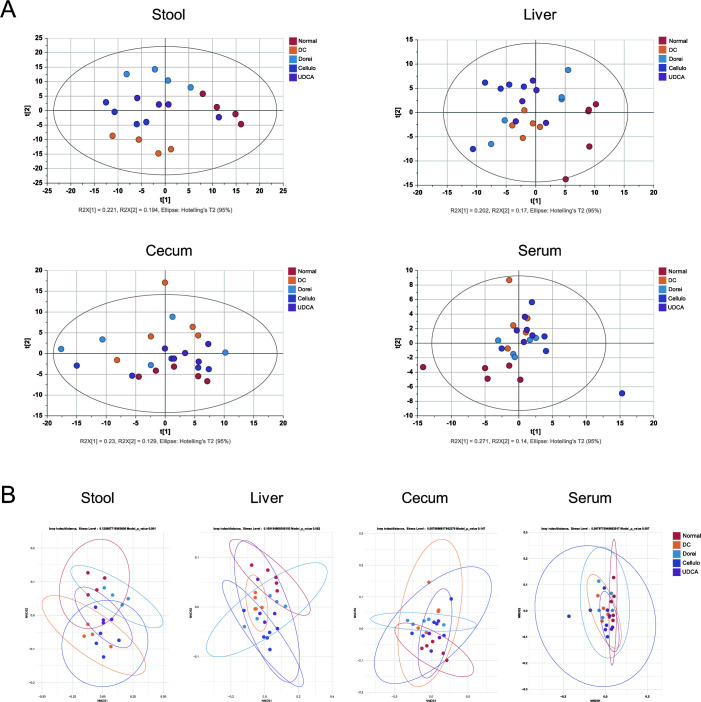
Unsupervised multivariate statistical analysis for stool, liver, cecum, and serum samples. (**A**) PCA of metabolite profiles of stool (R2X = 0.619, Q2 = 0.281), liver (R2X = 0.619, Q2 = 0.213), cecum (R2X = 0.622, Q2 = 0.176), and serum (R2X = 0.589, Q2 = 0.0984). The multivariate statistical analysis was performed by SIMCA 15. (**B**) NMDS (nonmetric multidimensional scaling) plot of metabolite profiles from stool (*P* = 0.001), liver (*P* = 0.002), cecum (*P* = 0.147), and serum (*P* = 0.007) samples based on PERMANOVA (permutational multivariate analysis of variance) using ggpubr function in ggplot2 package (version 0.4.0).

**Fig 4:**
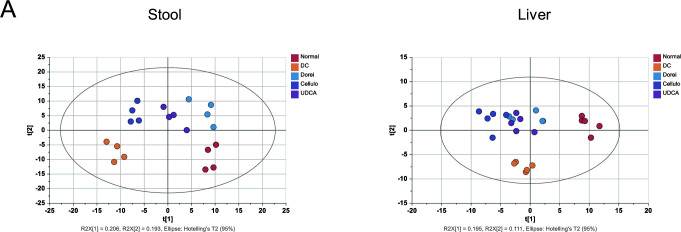
Supervised predictive model based on PLS-DA for stool (R2Y = 0.981, Q2 = 0.805) (A) and liver (R2X = 0.449, Q2 = 0.365) samples (B). The multivariate statistics was performed by SIMCA 15.

Multivariate statistics was applied to capture the characteristic profiles of treatment-specific metabolome of the stool, liver, cecum, and serum samples. Principal component analysis (PCA) for the stool samples demonstrated the most distinctive profiles according to the different groups, compared to the other biological matrix (R2X = 0.619 and Q2 = 0.281) (Fig. 3A). NMDS plot based on PERMANOVA confirmed the significant discrimination of the stool (*P* = 0.001) and liver samples (*P* = 0.003) (Fig. 3B). The profiles of cecum and serum metabolites were not clearly differentiated. Supervised multivariate statistics and partial least squares-discriminant analysis (PLS-DA) recapitulated the discrimination power of the fecal metabolome (R2Y = 0.981 and Q2 = 0.805) (Fig. 4A). The NC group, the treatment groups (Dorei, Cellulo, and UDCA groups), and the DC group were clearly distinguished by the t[1] vector. Likewise, the PLS-DA plot demonstrated the substantial discrimination of the hepatic metabolome, in which the NC group, the treatment groups, and the DC group were separated by the t[2] vector (R2Y = 0.449 and Q2 = 0.365) (Fig. 4B).

### Pathway analysis specify the treatment-dependent metabolic reprogramming

Considering the most distinctive profiles in the fecal and hepatic metabolome, we further interrogated the metabolic features, focusing on the two biological matrixes. Hierarchical clustering analysis (HCA) was conducted to probe each group-specific metabolic features. The top 50 metabolites were selected based on *P*-values from ANOVA, and the variable-wise clusters were constructed based on relative abundances and correlation.

**Fig 5:**
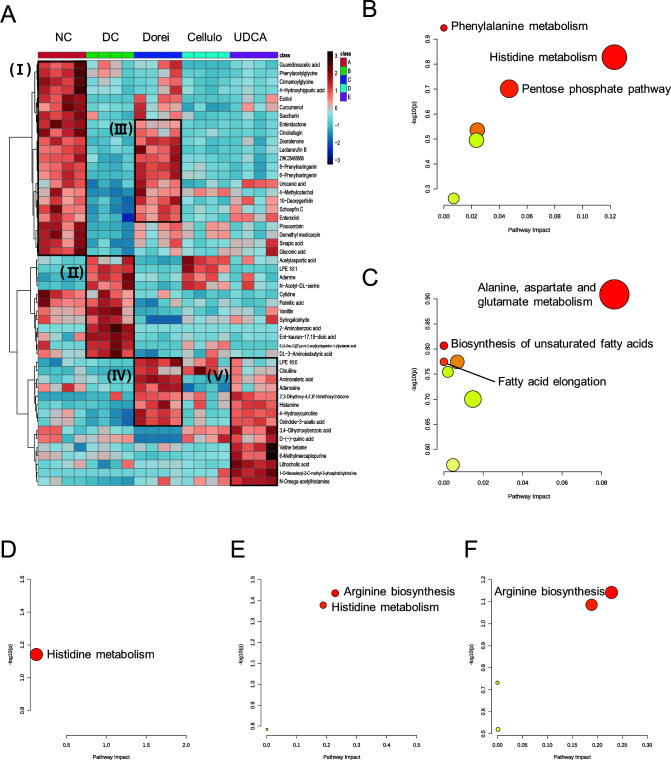
Stool metabolites that consist of co-regulated modules according to different treatments. (**A**) HCA of stool metabolic profiles based on Euclidean distance and Ward clustering algorithm. A heatmap includes top 50 metabolites following significant tests based on ANOVA. (B–F) Pathway over-representation analysis of metabolite modules that are clustered in panel A (**I–V**). *X*-axis represents the pathway impact values from pathway topology analysis; *y*-axis represents the level of pathway enrichment score arranged by −log(*P*-value). All analyses were conducted using MetaboAnalyst (version 4.0) (https://www.metaboanalyst.ca).

In stool samples, two clusters with highly upregulated metabolites were identified in NC (I) and DC (II) groups, respectively (Fig. 5A). In the Dorei group, the cluster of 12 metabolites (III) showed a similar pattern to the normal group while the Dorei group-specific cluster was identified, which included eight metabolites (IV). Cluster III, common with the NC group, included enterolactone, cinobufagin, zearalenone, lactarorufin B, ZINC35465958, 8-prenylnaringenin, 6-prenylnaringenin, urocanic acid, 4-methylcatechol, 10-deoxygerfelin, 2′,4′,6′-trihydroxydihydrochalcone, and enterodiol. Cluster IV, specific to the Dorei group, consisted of LPE 16:0, citrulline, 5-aminovaleric acid, adenosine, 2′,3-dihydroxy-4,4′,6′-trimethoxychalcone, histamine 4-hydroxyquinoline, and 2-oxindole-3-acetic acid. A cluster of upregulated metabolites was determined in the UDCA group (V), which shared the metabolites partially with the Dorei group.

Subsequent pathway over-representation analysis showed the metabolic specificity and commonality in stool samples. Cluster I pertained to partial enrichment of phenylalanine metabolism (raw *P* = 0.1136) and histidine metabolism (urocanic acid, *P* = 0.1487) in the NC group (Fig. 5B). Histidine metabolism is also marginally enriched in the Dorei group (histamine, *P* = 0.0722) and UDCA group (histamine, *P* = 0.0821), but not in the DC group (Fig. 5D). In addition, metabolic reprogramming was identified in arginine biosynthesis (*P* = 0.0367, *P* = 0.07216) in both Dorei and UDCA groups (Fig. 5E and F). The common metabolic pathways may be associated with the therapeutic effect of *B. dorei* and UDCA on liver cirrhosis. The DC group was featured by marginal upregulation of alanine-aspartate-glutamate metabolism, biosynthesis of unsaturated fatty acids, and fatty acid elongation (Fig. 5C).

**Fig 6:**
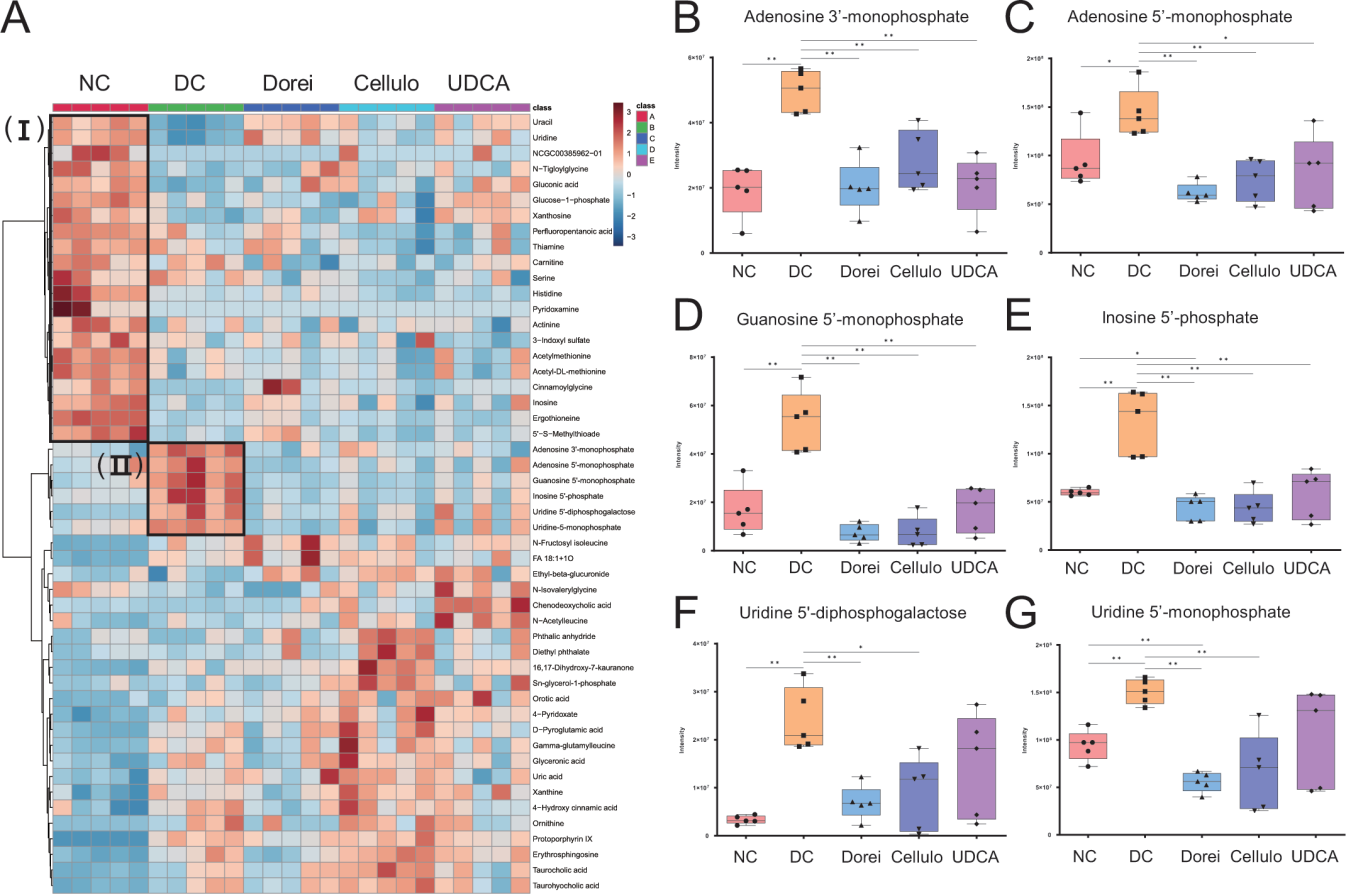
Hepatic metabolites that consist of co-regulated modules according to different treatments. (**A**) HCA of hepatic metabolites. A heatmap consists of metabolites that show significant mean differences (ANOVA) based on Euclidean distance and Ward clustering algorithm using MetaboAnalyst (version 4.0). (B–G) Relative abundances of metabolites in cluster II. Statistical significance is estimated by Kruskal–Wallis test (*P < 0.05 and **P < 0.01) using ggpubr function in ggplot2 package (version 0.4.0). Box-and-whisker plot is visualized by GraphPad Prism 9.

The liver metabolic profiles presented two distinctive clusters, belonging to the NC (I) and DC (II) groups (Fig. 6A). Beta-alanine metabolism (*P* = 0.0174), pyrimidine metabolism (*P* = 0.0555), thiamine metabolism (*P* = 0.0678), and vitamin B6 metabolism (*P* = 0.0678) were enriched in the NC group compared to the other groups. Cluster II showed significant enrichment for purine metabolism (*P* = 0.0107) in the DC group. Of note, purine metabolites (adenosine 3′-monophosphate, adenosine 5′-monophosphate, and guanosine 5′-monophosphate) were specifically upregulated in the DC group, whereas the levels in all treatment groups were sustained to the normal level (Fig. 6B through G).

### Key metabolites are identified from common metabolic signatures between the NC group and Dorei group against the DC group

**Fig 7:**
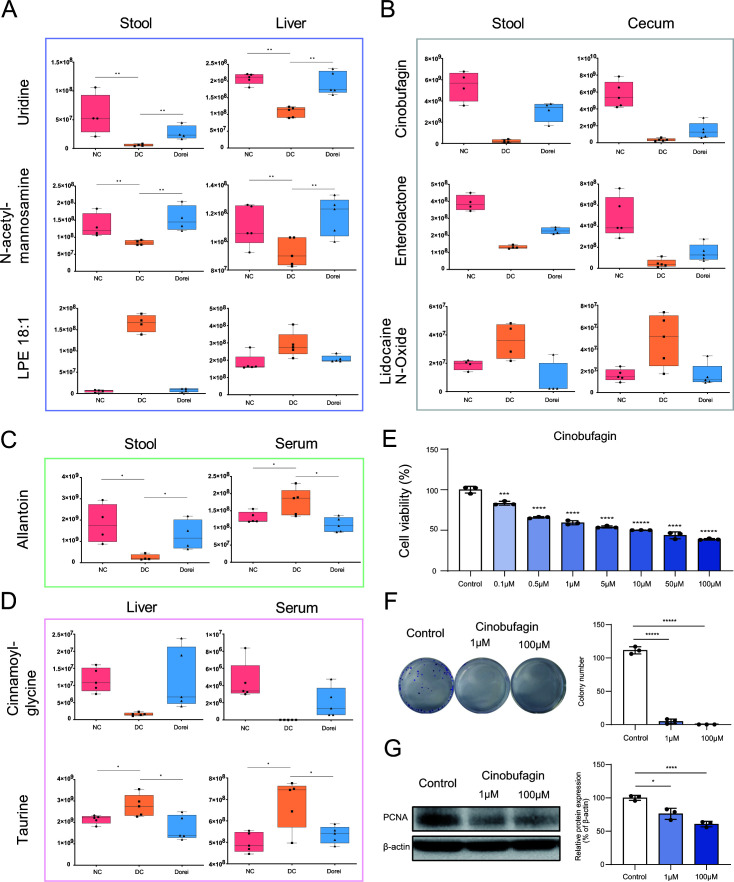
Identification of key metabolites from common metabolic signatures between the NC group and the Dorei group, compared to the DC group. (**A**) The relative abundances of metabolites that are similarly regulated in stool and liver samples for the NC and Dorei groups. (**B**) The relative abundances of metabolites that are similarly regulated in stool and cecum samples for the NC and Dorei groups. (**C**) The relative abundances of metabolites that are similarly regulated in stool and serum samples for the NC and Dorei groups. (**D**) The relative abundances of metabolites that are similarly regulated in liver and serum samples for the NC and Dorei groups. *Y*-axis represents the relative abundance. Statistical power was estimated by the false discovery rate(FDR)-adjusted *P*-values (*FDR < 0.25 and **FDR < 0.2) using ggpubr function in ggplot2 package (version 0.4.0). (**E**) The dose-dependent reduction of the HepG2 cell viability by cinobufagin based on MTT assay. (**F**) The reduced colony-forming ability of HepG2 cells by cinobufagin treatment. (**G**) The downregulation of PCNA by cinobufagin treatment based on Western blot analysis. Box-and-whisker plot and scatter plot with bar are visualized by GraphPad Prism 9 (statistical comparison was performed based on Student’s *t*-test *P < 0.05, **P < 0.01, ***P < 0.005, ****P < 0.0005, and *****P < 0.00005).

We further interrogated keystone metabolites that were similarly regulated between the NC and Dorei groups. Subsequently, the common metabolite levels were evaluated across the different samples (Fig. 7). The common features included metabolites that were significantly different in both the NC and the Dorei groups from the ones in the DC group (Fig. S4). Direction (e.g., up- or downregulation) was not considered since the opposite pattern may be involved in systemic translocation. In line with the multivariate statistics result, the stool sample showed the highest levels of exclusively common features (64% of fecal metabolites), which was followed by liver, serum, and cecum (Fig. S4).

Note that all keystone metabolites showed the same direction across different samples (Fig. 7A, B, and D) except allantoin (stool-serum) (Fig. 7C). The significantly higher metabolites were determined in NC and Dorei groups compared to DC group as follows: uridine and N-acetylmannosamine (stool, liver); cinobufagin and enterolactone (stool, cecum); cinnamoylglycine (liver, serum). For each set of selected metabolites, The receiver operating characteristic (ROC) area under the curve (AUC) was calculated each for NC and Dorei groups versus DC groups. Both uridine and cinnamoylglycine had the highest potential as biomarkers (AUC = 1) in both sample matrices.

N-acetylmannosamine, cinobufagin, and enterolactone also had high AUC values (AUC = 1) in the stool samples. Compared to NC and Dorei groups, LPE 18:1, lidocaine N-oxide, and taurine existed in higher amounts in DC groups. LPE 18:1 had the highest AUC values (AUC = 1) in the stool samples. Allantoin showed a significantly higher amount in normal and Dorei groups than in the DC group in stool samples, and a higher amount in the DC group than in normal and Dorei groups in serum samples. The metabolites aforementioned, cinobufagin, enterolactone, cinnamoylglycine, uridine, and N-acetylmannosamine may be linked to the healthy state, while lidocaine N-oxide and taurine may represent the state of liver cirrhosis. Further research is required to examine if the metabolites affect the liver or gut function causally.

For correction of multiple testing, FDR adjusted *P*-values were calculated following the Student’s *t*-test. Keystone metabolites taurine, allantoin, uridine, and N-acetylmannosamine passed the cutoff criteria of *P* = 0.25, and uridine and N-acetylmannosamine passed the cutoff criteria (14) of *P* = 0.2.

We selected four metabolites from each category that showed common patterns across different sample sources (Fig. S4). The metabolites included uridine, N-acetylmannosamine, cinobufagin, and cinnamoylglycine, which were found in relatively high abundances in the NC and Dorei groups compared to the DC group. Further *in vitro* experiments showed that among these metabolites, cinobufagin had significant inhibitory effects on HepG2 cells. HepG2 cells are often used as a model to study various aspects of liver diseases including liver cirrhosis (15, 16). Cinobufagin significantly inhibited cell viability in a dose-dependent manner (Fig. 7E), whereas uridine, N-acetylmannosamine, and cinnamoylglycine did not show any inhibitory effect (Fig. S5). Colony formation assay showed a significant reduction in the colony number of HepG2 cells upon treatment of cinobufagin in a dose-dependent manner (Fig. 7F). Additionally, the substantial decrease in the proliferation marker proliferating cell nuclear antigen (PCNA) implies that cinobufagin suppresses proliferation of HepG2 cells (Fig. 7G). The overall result shows that cinobufagin, a key metabolite that is upregulated in NC and Dorei groups, also has potential therapeutic effects in hepatic diseases such as liver cirrhosis.

### *B. dorei* treatment restores gut microbes linked to potential health benefits

16S rRNA gene amplicon sequencing of 86 human stool samples clearly confirmed changes in the gut microbiome of patients with alcoholic hepatitis and cirrhosis (Fig. S1 and S2), indicating the importance of restoring the gut microbial composition for alleviation of liver cirrhosis. The human gut microbiome analysis showed that *Bacteroides* showed a significant reduction in the Alcohol Cirrhosis group compared to the Normal Control (Fig. S1C). Also, the diversity analysis showed significant differentiation between the Normal Control and Alcoholic Cirrhosis groups (Fig. S2B and C).

**Fig 8:**
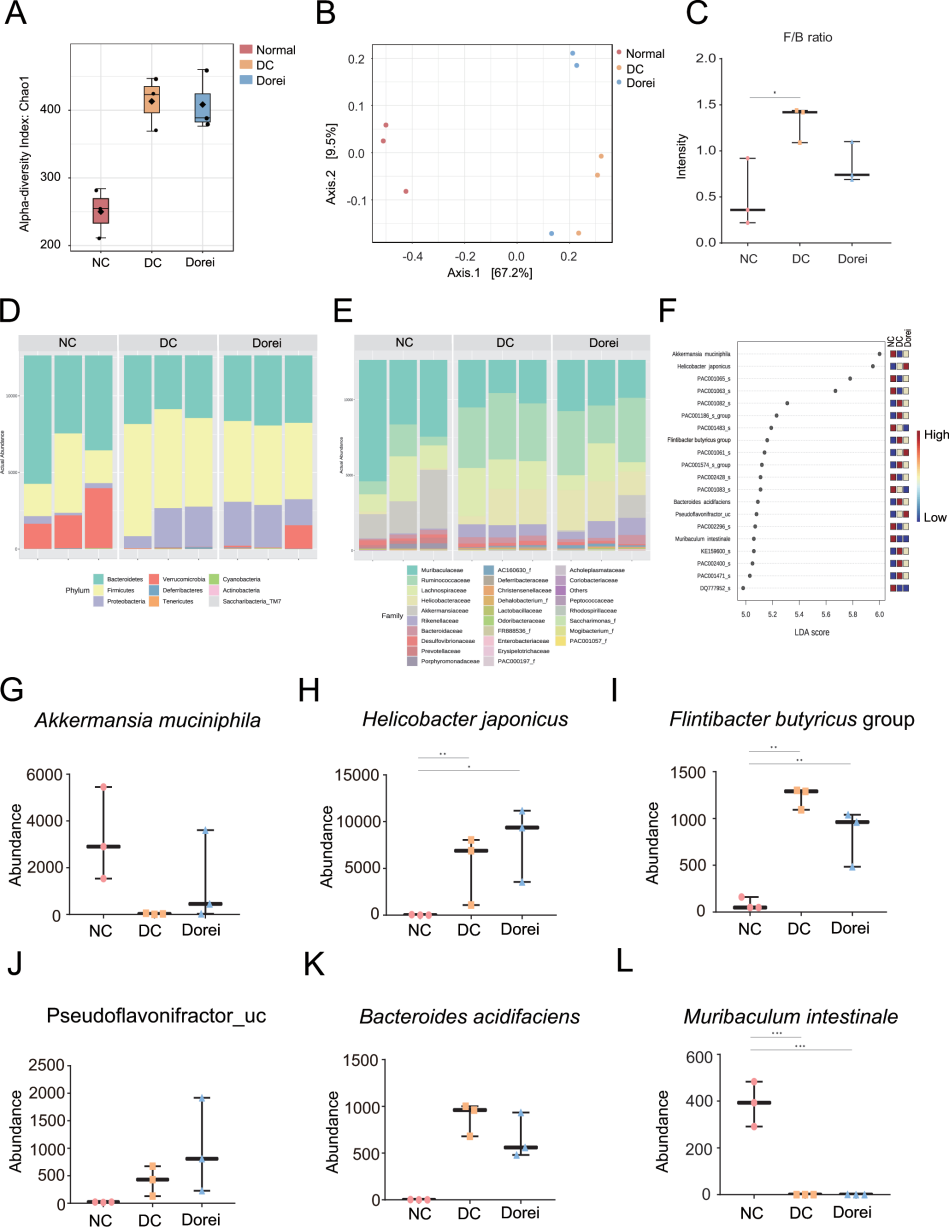
Taxonomic analysis of mouse gut-microbiome based on 16S rRNA gene amplicon sequencing. (**A**) Alpha diversity analysis on Chao1 for species richness estimation. (**B**) Principal coordinate analysis plots of beta diversity calculated based on the weighted UniFrac metric. (**C**) The F/B ratio. Stacked bar plot of microbial composition at the phylum level (**D**) and at the family level (**E**). (**F**) Taxonomic biomarker identification by linear discriminant analysis effect size analysis. (**G–L**) Relative abundances of the taxonomic biomarkers (*q* value < 0.3 and LDA score > 5.0). LEfSe analysis was performed by MicrobiomeAnalyst (version 2.0) (https://www.microbiomeanalyst.ca). Significant mean difference was estimated based on ANOVA with Benjamini-Hochberg adjustment (*P < 0.05, **P < 0.01, and ***P < 0.001) using ggpubr function in ggplot2 package (version 0.4.0). Box-and-whisker plot is visualized by GraphPad Prism 9.

16S rRNA gene amplicon sequencing data were also obtained from mouse stool samples, and the gut microbial composition was comparably analyzed among NC, DC, and Dorei groups. Unexpectedly, the NC group showed the lowest alpha diversity (Fig. 8A). Commonly, alpha diversity is lower in disease status; however, a few studies have proposed the reverse relation. Zeng et al. reported that fecal microbial diversity decreased from healthy to cirrhosis and increased from cirrhosis to early hepatocellular carcinoma (HCC) with cirrhosis (17). Beta diversity analysis showed clear discrimination of the compositional profiles of the three groups (Fig. 8B). The Firmicutes to Bacteroidetes (F/B) ratio was the highest in the DC group, whereas the ratios were marginally lower in the treatment groups (Fig. 8C). In the phylum level, each group showed different compositions as shown in Fig. 8D. The major difference was related to the relatively higher abundance of Verrucomicrobia in the NC group, while Proteobacteria was found in high proportion in DC and Dorei groups. The decrease of Bacteroidetes and increase of Proteobacteria and Firmicutes in the disease group show a similar pattern to the gut microbiome analysis of alcohol cirrhotic patients and normal control (Fig. S1A). However, the Verrucomicrobia phylum shows little change in the human gut microbiome, whereas it is reduced in the DC group of the DDC mouse model. NC, DC, and Dorei groups also showed different compositions at the family level. For example, *Muribaculaceae* and *Akkermansiaceae* were the most abundant in the NC group (Fig. 8E). *Ruminococcaceae*, *Lachnospiraceae*, *Helicobacteraceae*, and *Rikenellaceae* were relatively depleted in the NC group compared to DC group.

Of particular interest, *Akkermansia muciniphila*, a species in the Verrucomicrobia phylum, showed the highest LDA score. LDA score of linear discriminant analysis effect size (LEfSe) analysis determines the feature most likely to explain the difference between groups (LDA score > 5.0) (Fig. 8F). The abundance of *Akkermansia muciniphila* was the highest in the NC group, decreased in the DC group, and marginally sustained in the Dorei group (Fig. 8G). On the contrary, *Flintibacter butyricus* group and *Bacteroides acidifaciens* increased in DC group but decreased marginally when treated with *B. dorei* (Fig. 8I and K). *Helicobacter japonicus*, *Flintibacter butyricus* group, and *Muribaculum intestinale* showed significant difference between NC and DC groups, but not in DC and Dorei groups (Fig. 8H, I, and L). *Pseudoflavonifractor*_uc group increased marginally in the DC and Dorei groups compared to the NC group (Fig. 8J).

**Fig 9:**
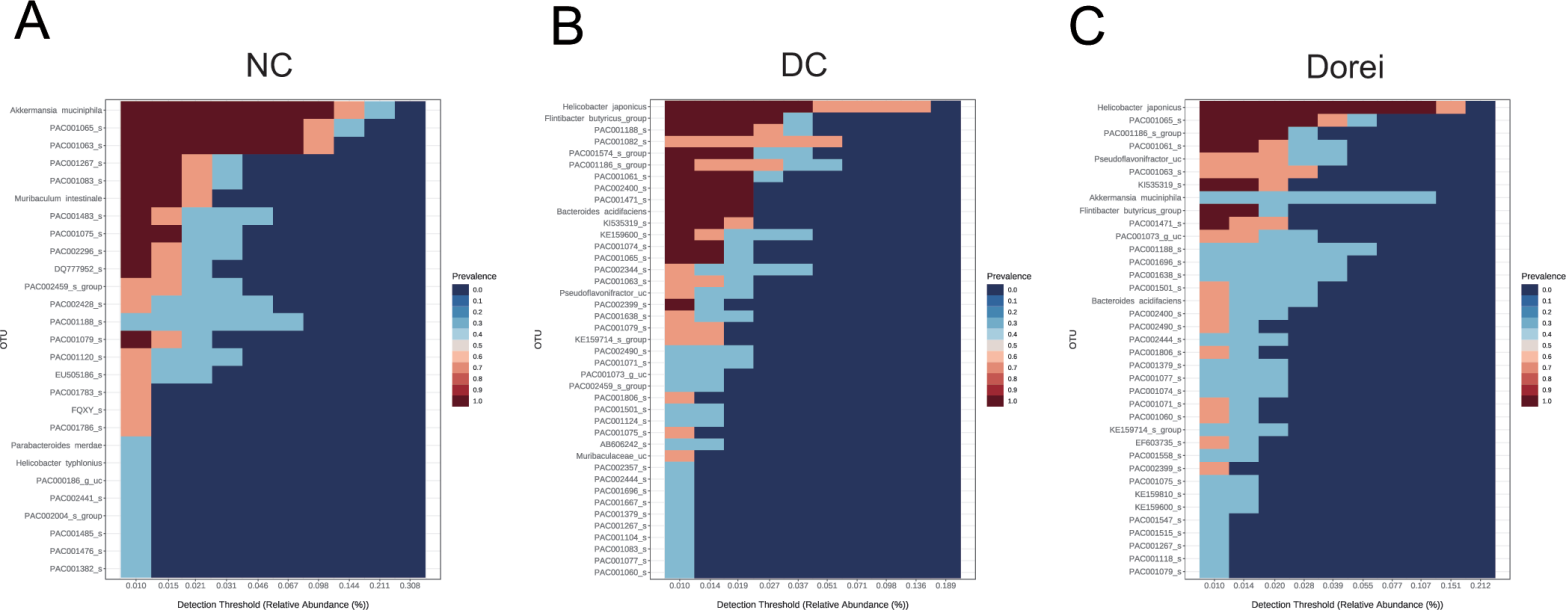
Identification of functionally crucial microbiota associated with each group. The core microbiome refers to the set of taxa that are detected in a high fraction of the population above a given abundance threshold. The count data are transformed to compositional (relative) abundances. Core microbiome analysis, implemented in MicrobiomeAnalyst (version 2.0), identifies unique and shared taxa for NC, DC, and Dorei groups based on a minimum 20% prevalence threshold with at least 0.01% detection threshold.

Core microbiome analysis determines the taxa that are shared among two or more microbial communities in a given host species or environment (18). The common taxa represent the most ecologically and functionally crucial microbiota associated with that host or environment (under the conditions sampled). At the species level in the NC group, *Akkermansia muciniphila* and *Muribaculum intestinale* showed the highest prevalence levels (Fig. 9). In the DC group, *Helicobacter japonicas*, *Flintibacter butyricus* group, and *Bacteroides acidifaciens* presented high prevalence. In the Dorei group, *Helicobacter japonicas*, *Pseudoflavonifractor*, *Akkermansia muciniphila*, and *Flintibacter butyricus* group showed high prevalence. Note that the prevalence of *Akkermansia muciniphila* was sustained in the Dorei group, but not the prevalence of *Muribaculum intestinale*.

## DISCUSSION

Our current investigation demonstrated that *B. dorei* treatment resulted in the significant amelioration of liver dysfunction in the liver cirrhosis mouse model. The functional improvement was conveyed by two characteristic metabolic clusters that pertained (i) the normalized levels to the NC group and (ii) the treatment-specific upregulation. Both metabolic features may indicate potential protective effects of the treatment on liver function dysregulated by DDC.

SCFAs showed the differential regulation across the different biological matrices. All six SCFAs showed consistent upregulation in the DC stool samples, while the opposite patterns were determined in the cecal samples. The decreased levels of cecal SCFAs in disease DC group recapitulate the common healthy effect in a healthy gut environment. The contrast pattern of the fecal SCFA content may represent the abnormal adsorption by dysfunctional receptors in the lumen (19), which may result in a higher rate of excretion in unhealthy subjects. The scenario is plausible considering the metabolic profiles in the ultimate target organ, the liver.

The conclusive interpretation of fecal metabolomic analysis is challenging. Nonetheless, one typical interpretation regards fecal metabolites as non-invasive biomarkers of gut diseases under the hypothesis that fecal metabolites represent the metabolites in the gut environment (20). Another perspective considers stool metabolites as excreted metabolites that are unabsorbed in the gut environment. Our result may be supported by the second scenario in which an unhealthy gut environment would dysregulate the receptors for the adsorption of SCFAs in the lumen, resulting in an increased abundance of SCFAs in stool samples.

The multicompartmental monitoring of metabolic changes provides direct information on systemic transportation and interactive effect, which helps in understanding the mode of action and elucidating the direct causality of the disease. Indeed, we identified the most distinctive metabolic changes by the treatment in two sample matrices, fecal and liver. Our study showed better predictive performance of fecal metabolites, compared to serum metabolites. Considering the close association between fecal metabolite and gut microbiome, key metabolites identified from stool samples have potential as noninvasive biomarkers, particularly for gut-liver axis-involved disease.

In fecal and liver samples, pathway over-representation analysis showed that among the Dorei treatment-specific metabolic signatures, metabolites involved in histidine metabolism may be directly associated with the liver protective effect dysregulated by DDC treatment metabolism is marginally enriched in the Dorei group and UDCA group, but not in the DC group. Histidine improves liver health by reducing lipogenesis (21), and the lower level of histidine is related to liver cirrhosis (22). The known function of histidine, together with the enriched urocanic acid and histamine in the NC group, suggests a positive role of histidine metabolism in liver health. The DC group showed marginal upregulation of alanine-aspartate-glutamate metabolism, biosynthesis of unsaturated fatty acids, and fatty acid elongation. Particularly, palmitic acid is the most cytotoxic fatty acid for the liver (23). The fatty acid causes hepatic fibrosis by activating hepatic stellate cells through inflammasomes and hedgehog signaling (24). Hyperactive activities of fatty acid metabolism in the DC group are a typical pathology, especially in the progression of hepatic fibrosis.

In liver samples, purine metabolites (adenosine 3′-monophosphate, adenosine 5′-monophosphate, and guanosine 5′-monophosphate) were specifically upregulated in the DC group, whereas the levels in all treatment groups were sustained to the normal level. The inhibition of purine metabolism has shown reduced HCC proliferation *in vitro* in the study of Chong et al. (25). This suggests that purine metabolism may be a promising therapeutic target for the progression of liver cirrhosis. Also, dysregulated purine and pyrimidine metabolisms have been reported for rats with dioscorea bulbifera rhizome (DBR)-induced liver damage (26). Similarly, phosphorylated pyrimidine and the sugar derivatives (uridine-5-monophosphate and uridine 5′-diphosphogalactose) showed significant upregulation in the DC group.

Keystone metabolites such as uridine, cinnamoylglycine, N-acetylmannosamine, cinobufagin, enterolactone, and cinnamoylglycine were found in high abundance in the NC and Dorei groups compared to the DC group. Uridine functions in the glycolysis pathway of galactose and improves hepatocellular fat accumulation and obesity (27). It also contributes to reducing cytotoxicity and suppressing drug-induced liver steatosis (28). Cinnamoylglycine has been presented as an indicator metabolite representing high microbiome diversity in serum (29). Cinnamoylglycine also represents colonization resistance for *Clostridium difficile*. In other words, it is a compound that is evaluated as a healthy gut microbiome marker that can inhibit pathogenic microorganisms. Conversely, a study shows that cinnamoylglycine increased in portal blood in high-fat diet treatment (30). Further studies are needed to confirm the direct effect of cinnamoylglycine on liver disease.

N-acetylmannosamine is a precursor of N-acetylneuraminic acid, which restores immunoglobulin G sialylation and maintains insulin sensitivity (31). Enterolactone is a type of postbiotics derived from lignin and has antioxidant, anti-inflammatory, anti-cancer, cardioprotective, and neuroprotective activities (32). Also, it protects against a variety of cancers, including breast, prostate, colorectal, lung, ovarian, endometrial, cervical cancers, and hepatocellular carcinoma (33). Cinobufagin is known to have an anti-tumor effect in liver cancer cells by inhibiting the aurora kinase A—mechanistic target of rapamycin—eukaryotic initiation factor 4E axis (34). Our findings also confirm the ability of cinobufagin to inhibit the proliferation of HepG2 cells. This validates the potential of cinobufagin as a therapeutic agent in hepatic diseases. Current research results have indicated the effects of cinobufagin through apoptosis activation (35), and further investigation is needed to understand the mechanisms underlying these effects of cinobufagin and its potential for therapeutic applications.

The metabolomic alteration by the treatment coincided with a significant compositional change of *Akkermansia muciniphila*, which is a potential microbe known to improve metabolic disorders associated with obesity, diabetes, liver diseases, and cardiometabolic disorders (36). *Akkermansia* is the only genus found in the gastrointestinal sample among the Verrucomicrobia phylum (37). *Akkermansia muciniphila* is known to take up 3% of healthy adult colon (38). The microbe is associated with a healthy intestine, and the abundance is negatively correlated with several diseases. *A. muciniphila* is reduced in patients with ulcerative colitis, Crohn’s disease, and type 2 diabetes (39, 40). The diseases weaken the integrity or reduce the thickness of the mucus layer, which may limit the major energy source for *Akkermansia muciniphila* (41). The species has been also reported for the preventive effect on fatty liver disease in the obese mouse model by controlling triglyceride (TG) synthesis in liver and by maintaining gut homeostasis (42). The report showed reduced levels of SREBP, a regulator of TG synthesis in liver tissue in the group treated with *Akkermansia muciniphila*. In addition, *Akkermansia muciniphila* administration has restored the F/B ratio and bacteria diversity in mice with a high-fat diet (43).

In this regard, our current study gives a systematic view of the potential therapeutic effect of *Bacteroides dorei* based on multiomics approaches such as metabolomic and microbiome analysis. The comprehensive profiles of specific metabolites of different matrices provide deeper insight into direct causality and mechanistic understanding. Of particular, we propose the potential key mediators of biotic and abiotic origin in the gut microenvironment.

Further research is required to confirm certain findings in our current study. Our results do not show significant increase in the relative abundance of *Bacteroides dorei* in the Dorei group at the time of necropsy. There may be several reasons for this, such as variability in individual gut microbiomes and colonization patterns. The most reasonable explanation for the phenomenon may be related to the timing of sample collection. The relative abundance of *B. dorei* in stool samples may have peaked earlier than the time of necropsy, and therefore, the observed lack of increase in relative abundance may be due to sample collection timing. A longitudinal study may be needed to observe the variation of the taxa abundance from strain treatment to necropsy in order to determine the underlying reasons for the lack of increase in *B. dorei* abundance in the stool samples of the Dorei group.

Also, further research should be focused on the mode of action of the metabolites that were significantly altered. The fact that our selected key metabolite demonstrates therapeutic effects in an actual *in vitro* model suggests its potential applicability for drug development in clinical trials. Metabolic benefits of potential pharmabiotics such as *B. dorei* need to be clarified by further *in vivo* experiments (44) (e.g., by clarifying the efficacy difference between wild-type strain and metabolite-deficient strain). This way, a specific gut microbiota-metabolite-liver pathway could be defined for the alleviation of liver cirrhosis, providing insights into the therapeutic mechanism targeting the gut microbiota. For future research, a comparative study of the therapeutic effect of (i) *B. dorei* only, (ii) keystone metabolites (e.g., cinobufagin) only, and (iii) *B. dorei* + keystone metabolites (e.g., cinobufagin) in *in vivo* analysis will contribute to a more specific proposal of a potential treatment strategy for liver cirrhosis.

Likewise, the direct association of the metabolites needs in-depth investigation coupled with the compositional characteristics of *Akkermansia muciniphila,* according to our microbiome analysis. The limitation of our study is the small sample sizes and discrepancies in sample numbers across different types of data, due to the challenges in obtaining sufficient samples for multiple analyses. For future studies, a collection of larger sample sizes is likely to give more robust evidence in metabolomics and microbiome research. Further study on the correlation of metabolomics and microbiome data is needed for comprehensive investigation and application of gut microbiota-targeted mechanisms.

## MATERIALS AND METHODS

### Strain preparation

The *Bacteroides* strain was provided by CJ Bioscience, Inc., Seoul, South Korea. The strain was spread on a solid Reinforced Clostridial Medium and cultured at 37°C for 48 hours under anaerobic conditions. The strain was adjusted at a concentration of 10^9^ CFU per 200 µL after incubation.

### DDC mouse model

The following procedure was performed by Hallym University College of Medicine, Chuncheon, South Korea. Pathogen-free 5-week-old C57BL/6 mice were purchased from Dooyeol Biotech (Seoul, South Korea). Liver cirrhosis in mice was induced by DDC diet. Five-week-old C57BL/6 mice were divided into five groups (*n* = 5/group; normal, DDC, *B. dorei*, *B. cellulosilyticus*, and UDCA). All groups were fed the normal diet during the adaptation period of 1 week, after which the normal group continued to eat the normal diet for 3 weeks, and the other groups ate the DDC diet for 3 weeks. Bacterial strains were given a DDC diet twice a week (10^9^ CFU/200 µL, 3 weeks).

### Animal sacrifice

The following procedure was performed by Hallym University College of Medicine, Chuncheon, South Korea. The animals were sacrificed via inhalation anesthesia overdose (isoflurane, Aerane; Baxter, Deerfield, IL, USA) at the end of the treatment period. They were weighed, and stool, liver, cecum, serum, and colon were collected. Whole blood (800 µL) samples were centrifuged (19,000 × *g* for 5 min) to collect serum. Stool, liver, cecum, and colon were rapidly excised and stored at −80°C.

### Serum biochemistry

The blood was collected from sacrificed control or treated mice. The collected whole blood was centrifuged at 2,000 × *g* for 20 min to collect serum and stored at −80°C. T-BIL was quantified in animal serum using a biochemical blood analyzer (KoneLab 20, Thermo Fisher Scientific, Waltham, MA, USA).

### RNA extraction and quantitative real-time reverse-transcription polymerase chain reaction

Total RNA was extracted from the liver tissue using a High Pure RNA Isolation Kit (Roche, Mannheim, Germany). Aliquots of total RNA (2 µg) were transformed into cDNA using a cDNA reverse transcription kit (Applied Biosystems, Foster City, CA, USA). The cDNA subsequently underwent amplification for quantitative qPCR using Luna Universal Probe qPCR Master Mix (New England Biolabs, Beverly, MA, USA) and target-specific probe primer (Applied Biosystems, Foster City, CA, USA) for COL1A1, TGF-β, and IL-10. Relative expression was calculated using comparative ∆∆Ct values.

### Untargeted metabolomic analysis using LC-Orbitrap MS

Experiments were performed in five replicates (liver, cecum, and serum samples) and four replicates (stool samples) and analyzed in random order. Fecal samples excreted from mice were collected and used for untargeted metabolomic analysis. Due to the limited amount of fecal samples, we performed the analysis on four biological replicates per group for pre-processing and sample analysis. Stool, liver, and cecum samples (100 mg) were extracted by solvent mixture 1 (1.2 mL for stool and cecum samples, 1.1 mL for liver samples, acetonitrile: water, 1:1, vol/vol) and solvent mixture 2 (0.7 mL for stool and cecum samples, 0.6 mL for liver samples, acetonitrile: methanol, 1:3, vol/vol) for metabolomic profiling. Serum samples (100 µL) were extracted by solvent mixture (3.9 mL, methanol: acetonitrile: water, 3:3:2, vol/vol/vol). After 5 min of sonication and centrifugation each, the aliquot (160 µL) was transferred to a new vial and concentrated to complete dryness using a speed vacuum concentrator (SCANVAC, Seoul, South Korea). The dried sample was re-constituted with 70% ACN (acetonitrile) (70 µL) for mass-spectrometric analysis.

Samples were chromatographically separated by an UltiMate-3000 UPLC system (Thermo Fisher Scientific, Waltham, MA, USA) equipped with 150 × 2.1  mm UPLC BEH 1.7-µm C18 column (Waters, Milford, MA, USA) and 5.0 mm × 2.1 mm UPLC BEH 1.7-µm C18 VanGuard Pre-Column (Waters, Milford, MA, USA). The mobile phase consisted of solvent A (water with 0.1% formic acid) and solvent B (100% acetonitrile with 0.1% formic acid). The flow rate was set to 300 µL/min, and the gradient of solvent B was programmed as follows: 0–2 min, 3%; 2–11 min, 3%–100%; 11–12 min, 100%; 12–15 min, 3%.

Mass-spectrometry was conducted in polarity-switching ionization mode using Q-Exactive plus Orbitrap (Thermo Fisher Scientific, Waltham, MA, USA) with a heated electrospray ionization probe. Full mass spectrum (MS) scan was operated in the range of 70–1,000 *m*/*z* (resolution: 70,000 FWHM at *m*/*z* = 200) with automatic gain control target of 1e6 ions and maximum injection time of 100 ms. Tandem mass spectra (MS/MS) were collected on pooled samples by each ionization mode. Data acquisition and pre-processing were conducted using Xcalibur software (Thermo Fisher Scientific, San Jose, CA, USA). RAW data files obtained from LC-Orbitrap MS were processed using MS-DIAL software (version 4.7, Thermo Fisher Scientific, San Jose, CA, USA).

### Cell culture

HepG2 cell line was obtained from American Type Culture Collection (Manassas, VA, USA) and was maintained in minimum essential medium (Gibco, Grand Island, NY, USA) supplemented with 10% fetal bovine serum (Biowest, Riverside, MO, USA) and 1% penicillin/streptomycin (Gibco) at 37°C and 5% Co_2_ incubator.

### Cell viability assay

HepG2 cells (5 × 103 cells/96-well plate) were incubated for 24 hours, and starvation was performed for 4 hours. Uridine, N-acetylmannosamine, cinobufagin, and cinnamoylglycine were purchased from Sigma (St. Louis, MO, USA) and dissolved in dimethyl sulfoxide (DMSO). HepG2 cells were treated with different concentrations of uridine, N-acetylmannosamine, cinobufagin, and cinnamoylglycine for 24 hours, and then incubated with 3-(4, 5-dimethyl-2-thiazolyl)−2, 5-diphenyltetrazolium bromide (MTT, Sigma Aldrich) for 4 hours at 37°C and 5% Co_2_ incubator. The formazan crystals were dissolved in DMSO and their absorbance was measured at 570 nm using a microplate reader (Molecular Devices, Silicon Valley, CA, USA).

### Colony formation assay

HepG2 cells (5 × 102 cells/12-well plate) were incubated for 24 hours and then treated with different concentrations for 2 weeks. The media was replaced every 3 days. After treatment, the cells were fixed with methanol and stained with 0.5% crystal violet. The quantification analysis of colony formation in each group was performed using ImageJ software (v 1.54f, https://imagej.nih.gov).

### Western blot

After treatment, the protein was extracted with a radioimmunoprecipitation assay buffer (Thermo Scientific, Waltham, MA, USA). After that, cell lysates were analyzed by bicinchoninic acid assay for evaluated protein concentrations. Cell lysates were loaded on a 10% sodium dodecyl sulfate-polyacrylamide gel for electrophoresis and then transferred to the polyvinylidene fluoride (PVDF) membrane. The PVDF membrane was blocked for 1 hour with 5% bovine serum albumin solution and incubated with primary antibodies against PCNA (1:1,000) and β-actin (1:2,000) overnight. Subsequently, it was incubated for 1 hour with anti-mouse secondary antibody (1:2,000) or anti-rabbit secondary antibody (1:2,000). Finally, PVDF membrane was detected by using Clarity Western Enhanced chemiluminescence solution (Bio-Rad, Hercules, CA, USA) and determined by ChemiDoc imaging system (Bio-Rad). Primary antibodies, PCNA, and β-actin were purchased from Cell Signaling Technology (Danvers, MA, USA) and secondary antibodies were purchased from Santa Cruz Biotechnology (Dallas, TX, USA).

### Patients

A prospective cohort study was conducted between April 2017 and March 2020 (ClinicalTrials.gov NCT04339725). This study was conducted in patients with liver disease who were followed up at Hallym University. A total of 86 subjects were enrolled and analyzed, consisting of healthy control (*n* = 22), alcohol hepatitis (*n* = 28), and alcohol cirrhosis (*n* = 36). Patients received standard care for their disease regardless of study enrollment. For health control, we enrolled normal population coming to the hospital for a medical examination. The alcohol hepatitis and alcohol cirrhosis groups enrolled patients with elevated liver enzyme levels (AST > 50 IU/L, ALT > 50 IU/L). Alcohol cirrhosis was diagnosed based on the presence of complications (varix, ascites, and encephalopathy), blood tests, imaging findings, FibroScan, or pathological liver results. Patients with a history of severe alcoholic hepatitis receiving steroid treatment, viral hepatitis, pancreatitis, Wilson’s disease, cancer, autoimmune hepatitis, hemochromatosis, or drug-induced liver damage were excluded. This study was pursued by the ethical guidelines of the 1975 Helsinki Declaration, which was reflected in the prior approval of the institutional review notice of human research in the hospital received from each participant. Baseline assessments were conducted for liver function tests, complete blood counts, and viral markers. Patients with alcohol hepatitis and alcohol cirrhosis underwent abdominal ultrasound of computed tomography. Serum biochemical parameters included aspartate aminotransferase (AST), alanine transaminase (ALT), gamma-glutamyl transpeptidase, creatine, TG, cholesterol, and high-density lipoprotein cholesterol. Enrolled patients and controls underwent clinical analysis and stool sampling. The clinical data were consistent with the 16S rRNA gene amplicon sequencing data; the control group took stool samples during medical examination, and the patient obtained the stool box at the time of admission to the hospital and stored it in a −80°C refrigerator.

### Statistical analysis

Multivariate statistical analysis (e.g., PCA and PLS-DA) was performed using SIMCA 15 (Umetrics AB, Umea, Sweden). Heatmap was generated based on the Euclidean distance and Ward clustering algorithm, and pathway over-representation analysis was performed in MetaboAnalyst (https://www.metaboanalyst.ca/ accessed on 20 April 2022). NMDS plots and box and whisker plots were constructed using ggpubr function in ggplot2 packages (R version 4.0.2 and RStudio version 1.3.959). FDR-adjusted Student’s *t*-test and one-way ANOVA followed by the Benjamini-Hochberg procedure were also performed using ggpubr function in ggplot2 packages in R. One-way ANOVA followed by Tukey’s *post hoc* test was performed in GraphPad Prism 9. Stacked bar plot, heatmap, LEfSe, pie charts, and core microbiome analysis were created and performed in MicrobiomeAnalyst (https://www.microbiomeanalyst.ca/ accessed on 25 April 2022). Diversity analysis was statistically estimated by the Wilcoxon rank-sum test in EzBioCloud (https://www.ezbiocloud.net/). LEfSe analysis of 16S rRNA amplicon sequencing data and functional biomarker analysis based on KEGG pathway categories were also performed in EzBioCloud. In detail, functional profiles were predicted in EzBioCloud based on the reference genome database and MTP taxonomic profile data, which contain information on gene functions for each species. Then, PICRUSt and MinPath were used to predict modules and pathways. PICRUSt identifies modules and pathways based on the estimated functional profile, while MinPath uses pre-calculated module and pathway information for each species based on the reference genome database to provide analysis results.

## SUPPLEMENTAL MATERIAL

Fig. S1 spectrum.05349-22-s0001.eps Human gut microbiome taxonomic analysis based on 16S rRNA gene amplicon sequencing.

Fig. S2 spectrum.05349-22-s0002.eps Taxonomic biomarker, diversity, and functional biomarker analysis.

Fig. S3 spectrum.05349-22-s0003.eps Evaluation of the protective effects of strain supplementation and UDCA on liver fibrosis induced by the DDC diet.

Fig. S4 spectrum.05349-22-s0004.eps Prioritization of key metabolites based on identification of co-occurrent metabolites across different sample matrices.

Fig. S5 spectrum.05349-22-s0005.eps HepG2 cell viability by dose-dependent treatment of N-acetylmannosamine, cinnamoylglycine, and uridine based on MTT assay.

Supplemental methods spectrum.05349-22-s0006.docx Targeted analysis of short chain fatty acids using LC-Orbitrap MS; stool sample collection and 16S rRNA gene amplicon sequencing analysis.

ASM does not own the copyrights to Supplemental Material that may be linked to, or accessed through, an article. The authors have granted ASM a non-exclusive, world-wide license to publish the Supplemental Material files. Please contact the corresponding author directly for reuse.
