# Supplementary material for: Systemic multiomics evaluation of the therapeutic effect of Bacteroides species on liver cirrhosis in male mice
Source: Microbiol Spectr. 2023 Oct 11;11(6):e05349-22. doi: 10.1128/spectrum.05349-22 (PMC10848840; doi:10.1128/spectrum.05349-22)
Supplement: Supplemental methods — Targeted analysis of short chain fatty acids using LC-Orbitrap MS; stool sample collection and 16S rRNA gene amplicon sequencing analysis. [file spectrum.05349-22-s0006.docx]

**Systemic Multiomics Evaluation of Therapeutic Effect of *Bacteroides* Species on Liver Cirrhosis in Male Mice**

**SUPPLEMENTARY METHODS**

**Targeted analysis of short chain fatty acids using LC-Orbitrap MS.**

The analysis was performed according to our previous method^1^. The supernatant (40 µL) of solvent mixture 1 (acetonitrile: water, 1:1, v/v) was mixed with 20 µL of 200 mM 3-nitrophenylhydrazine (3NPH)-HCL in 70% acetonitrile and 20 µL of a 1-ethyl-3-(3-dimethylaminopropyl) carbodiimide (EDC)-HCL (120 mM) dissolved in 6 % pyridine solution. The mixed solution was incubated for 30 min at 40 °C and diluted with 1.92 ml of 70 % acetonitrile.

Liquid chromatography of the diluted derivatives was performed on Ultimate-3000 UPLC system (Thermo Fisher Scientific, Waltham, MA, USA) and a 150×2.1 mm UPLC BEH 1.7-μm C18 column (Waters, Milford, MA, USA) equipped with 5.0mm ×2.1mm UPLC BEH 1.7 μm C18 VanGuard Pre-Column (Waters, Milford, MA, USA). The mobile phase consisted of solvent A (water with 0.01% formic acid) and solvent B (100% acetonitrile with 0.01% formic acid) with a flow rate of 300 µL/min. The gradient of solvent B was programmed as follows: 0–2 min, 15%; 2–11 min, 15-55%; 11–12 min, 100%; 12–15 min, 15%. For both MS1 and MS/MS analysis, injection volume was set to 2 µL. Mass spectra were acquired using liquid chromatography based Orbitrap mass spectrometry in a Q-Exactive Plus instrument (Thermo Fisher Scientific, Waltham, MA, USA), equipped with an electrospray ionization (ESI) interface (HESI-II) in negative ionization. The system was controlled using Xcalibur 4.0 and Q-Exactive Tune software. Data processing for targeted analysis was performed in Tracefinder software (version 4.0, Thermo Fisher Scientific, San José, CA, USA). A mass tolerance for precursor ion and retention time tolerance were set to 5 ppm and 0.5 min, respectively.

**Stool sample collection and 16S rRNA gene amplicon sequencing analysis.**

Genomic DNA for 16S rRNA gene amplicon sequencing was extracted with a QIAamp stool kit (Qiagen, Germany) and library was prepared with a NEBNext Ultra Ⅱ FS DNA Library Prep Kit for Illumina (New England BioLabs, USA) according to the manufacture’s instructions. The quantification of libraries was checked using a Qubit dsDNA HS assay kit (ThermoFisher Scientific, USA) and confirmed by qPCR with KAPA SYBR FAST qPCR Master Mix kit (Kapa Biosystems, USA), The quality of libraries was assessed on a Bioanalyzer 2100 (Agilent, USA) using a DNA 12000 chip. All libraries were sequenced on the NovaSeq 6000platform (Illumina, USA) with a paired end (PE) 150 bp reads.

The analysis was performed according to our previous method^2^. Briefly, DNA was extracted using QIAamp stool kit and amplification of the V3 - V4 region of the bacterial 16S rRNA gene was performed using barcoded fusion primers. The target of 16S rRNA gene was amplified using fusion primers 341F (5’ – AATGATACGGCGACCACCGAGATCTACAC-XXXXXXXX-TCGTCGGCAGC GTCAGATGTGTATAAGAGACAG-CCTACGGGNGGCWGCAG -3’; underlining indicates the target region primer and X indicates the barcode region) and 805R (5’ CAAGCAGAAGACGGCATA CGAGAT-XXXXXXXX-GTCTCGTGGGCTCGGAGATGTGTATAAGAGACAG-GACTACHVG GGTATCTAATCC -3’), in which included sequencing adapters and dual-index barcodes of the Nextera XT kit (Illumina, San Diego, CA, USA). The amplification was performed in the C1000 touch thermal cycler polymerase chain reaction system (Bio-Rad Laboratories, Inc., USA) with the following conditions; initial denaturation of 3 min at 95 °C; followed by 25 cycles of denaturation at 95 °C for 30s, annealing at 55 °C for 30s, extension at 72 °C for 30s and final extension at 72 for of 5 min. Each amplified PCR product was confirmed with 1% agarose gel electrophoresis and visualized on a Gel Doc XR+ imaging system (Bio-Rad laboratories, Inc., USA). The amplified products were purified, and size selected by Agencourt AMPure XP beads (Beckman Coulter, USA). Library was constructed from pooled PCR products and quality of library was assessed on a Bioanalyzer 2100 (Agilent, USA) using a DNA 12000 chip and quantified by qPCR with KAPA SYBR FAST qPCR Master Mix kit (Kapa Biosystems, USA). Sequencing was performed according to the manufacture’s instructions reagent kit V3 in PE250 bp mode.

Microbiome taxonomic profiling of human stool microbiome was conducted database version PKSSU4.0. After taxonomic profiling of each sample, comparative microbiome taxonomic profile (MTP) analyzer of EZBioCloud was used for the comparative analysis of the samples. The number of operational taxonomic units (OTUs) picking was Good’s coverage, rarefaction, and alpha-diversity indices including ACE, Chao1, Jackknife, Shannon, Simpson, and NPShannon were calculated. Beta-diversity was shown by clustering using the unweighted pair group method with arithmetic mean (UPGMA) and principal coordinate analysis (PCoA).

Mice colon contents analysis for 16S rRNA amplicon sequencing was carried out in 3 replicates each for NC, DC, Dorei, and Cellulo groups (Department of Pathology, Hallym University College of Medicine, Chuncheon, Republic of Korea). Mouse colon contents were used for 16s rRNA amplicon sequencing. Due to the limited amount of the colon contents, we performed the analysis on 3 biological replicates per every group for pre-processing and sample analysis. The illumina paired-end data as 2 FASTQ files were uploaded to the EzBioCloud 16S-based MTP app (CJ Bioscience Inc., Seoul, Korea) to check the data quality. EzBioCloud MTP pipeline was employed for diversity estimation using PKSSU4.0 version database and Open reference UCLUST_MC2 for OTUs picking at 97% cut-off. The MTP app was used to detect and filter out sequences of low quality with regard to read length (<100 bp or >2000 bp) and averaged Q values less than 25. Microbial richness was measured by Chao1 and the number of OTUs found in the MTP index. Alpha diversity was measured by Chao1 index, and principal coordinate analysis (PCoA) plot was drawn for beta diversity analysis calculated using the weighted UniFrac metric.

**REFERENCES**

1. Seok Yu J, Soo Youn G, Choi J, Kim CH, Yong Kim B, Yang SJ, et al. Lactobacillus lactis and Pediococcus pentosaceus-driven reprogramming of gut microbiome and metabolome ameliorates the progression of non-alcoholic fatty liver disease. Clinical and Translational Medicine. 2021 Dec 1;11(12):e634.
2. Song HS, Whon TW, Kim J, Lee SH, Kim JY, Kim YB, et al. Microbial niches in raw ingredients determine microbial community assembly during kimchi fermentation. Food Chemistry. 2020 Jul 15;318:126481.
